# Direct Extraction and Determination of Free Nicotine in Cigarette Smoke

**DOI:** 10.1155/2024/9273705

**Published:** 2024-05-04

**Authors:** Li Li, Jing Wen, Yuyang Deng, Juan Yang, Yue Yuan, Yi Shen, Guoce Liu, Yonghong Tian, Dean Lei

**Affiliations:** Technology Center, China Tobacco Sichuan Industrial Co., Ltd., No. 56, Section 1 of Cheng Long Avenue, Jinjiang District, Chengdu 610066, China

## Abstract

The accurate determination of the free nicotine content in cigarette smoke is crucial for assessing cigarette quality, studying harm and addiction, and reducing tar levels. Currently, the determination of free nicotine in tobacco products primarily relies on methods such as pH calculation, nuclear magnetic resonance (NMR) spectroscopy, headspace solid-phase microextraction (HS-SPME), and traditional solvent extraction. However, these methods have limitations that restrict their widespread application. In this study, the free nicotine in cigarette smoke was directly extracted by using cyclohexane according to the traditional solvent extraction method and detected via gas chromatography-mass spectrometry. Compared with the traditional two-phase solvent extraction, our experimental method is easy to execute and eliminates the influence of aqueous solutions on the original distribution of nicotine in cigarette smoke particulate matter. Furthermore, the presence of protonated nicotine in tobacco does not affect the determination. Compared with HS-SPME and NMR spectroscopy, our approach, which involves solvent extraction followed by chromatographic separation and instrumental detection, offers simplicity, improved precision, better detection limits, and reduced interference during the instrumental detection stage. The standard addition recoveries in the conducted experiment ranged from 96.2% to 102.5%. The limit of detection was 2.8 *μ*g/cig, and the correlation coefficient (*R*^2^) for the quadratic regression of the standard curve exceeded 0.999. The relative standard deviation for parallel samples was between 1.7% and 3.4% (*n* = 5), fully meeting the requirements for the determination of free nicotine in cigarette smoke. Analysis of cigarette samples from 38 commercially available brands revealed that the content of free nicotine ranged from 0.376 to 0.716 mg/cig, with an average of 0.540 mg/cig, and free nicotine accounted for 39.1%–88.8% of the total nicotine content.

## 1. Introduction

Nicotine is the most important characteristic component of tobacco. When smokers smoke cigarettes, nicotine can stimulate and excite the peripheral and central nervous systems of smokers and produce signals. Therefore, nicotine is closely related to smokers' sensory experience, physiological feelings, and smoking addiction. Studies have shown that the sensory experience and physiological feeling of smoking cigarettes with high nicotine content are not necessarily strong, and the strength and impact of smoking cigarettes with low nicotine content are not necessarily weak [1–3]. Sometimes, the nicotine content in the mainstream smoke of different cigarettes is similar or even the same, but the strength and impact of smoking are very different, mainly due to the different forms of nicotine in cigarette smoke [4, 5].

**Figure 1:**
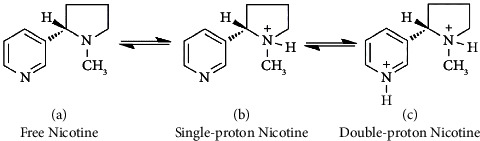
Three forms of nicotine.

Nicotine is a weak binary base composed of a pyridine ring and a hydrogenated pyrrole ring. When in contact with acidic components, it can capture at most two protons and turn into salts. Therefore, nicotine can exist in three forms in tobacco: the free state, monoprotonated state, and diprotonated state (Figure 1). Free nicotine is characterized by high lipophilicity, making it easy to penetrate human oral mucosa and be absorbed [6]. It has a strong physiological effect on the central nervous system, and cigarettes with high free nicotine content are characterized by high strength and impact [7]. Protonated nicotine features high hydrophilicity, resulting in slow absorption and metabolism in the human body [8]. The protonation state of nicotine also affects its transfer rate to cigarette smoke. Compared with protonated nicotine, free nicotine is more volatile and has a higher transfer rate to the smoke, which significantly affects the strength, impact, and irritancy of cigarettes [1, 9]. Therefore, it is crucial to accurately determine the contents of the different forms of nicotine in tobacco, particularly free nicotine from cigarette smoke. This is essential for the quality assessment of cigarettes and tobacco leaves, for research on the harm and addiction caused by cigarettes, and for studies on reducing the tar content of cigarettes.

Currently, the analysis of nicotine in tobacco primarily focuses on determining the total amount of the three forms of nicotine, while the determination of free nicotine content is rarely conducted. The main reason for this is that the method for determining the total nicotine content in tobacco is relatively easy. It involves acidizing (converting all forms of nicotine in tobacco into protonated nicotine) to accurately determine the total nicotine content in tobacco through different instrumental analysis methods, [10] alkalization (converting all nicotine in tobacco into free nicotine and then measuring), [11] or direct solvent extraction (using a solvent with good solubility to extract all forms of nicotine into the solution and then testing) [12, 13]. Several methods exist for determining free nicotine content, such as pH calculation, [5, 13–15] nuclear magnetic resonance (NMR) spectroscopy, [1, 16–19] headspace solid-phase microextraction (HS-SPME), [20–23], and solvent extraction [15, 24]. However, each method has limitations and shortcomings that restrict its application.

The pH calculation method involves measuring the total amount of different forms of nicotine in tobacco, preparing a solution of a certain concentration, determining the pH value of the solution, and then calculating the free nicotine content using the Henderson–Hasselbalch equation. The disadvantage of this approach is that the samples analyzed are generally solid or solid-like heterogeneous systems. After being dissolved as a solution, the original form of nicotine in the sample undergoes changes owing to the introduction of water and the influence of other acidic or alkaline components in tobacco. Consequently, calculating the free nicotine content in the solution at this pH value and considering it as the actual amount of free nicotine in the sample is not accurate. The calculated results may significantly differ from the original amount, and this approach has also faced opposition from some scholars [5, 17, 25].

NMR spectroscopy is an absorption spectrometric analysis method that utilizes the NMR spectra of substances for structure determination and qualitative and quantitative analysis. This method has been applied to determine free nicotine content in electronic cigarette liquids, [17] aerosols from heat-not-burn tobacco products, [18] and particulate matter from conventional cigarette smoke [16]. However, there are also some limitations in using NMR spectroscopy for determining free nicotine content in tobacco, in terms of selectivity, resolution, baseline, and detection limit [15].

The HS-SPME method is based on the difference in volatility between free and protonated nicotine. However, it cannot effectively verify the complete separation of free and protonated nicotine or any transformation that occurs during the measurement. The water in tobacco, solid-phase microextraction temperature, and solvent used to dissolve the internal standard significantly impact the accuracy and precision of the method [26]. Some scholars have also utilized ultraviolet-visible spectroscopy to determine the free nicotine content in e-cigarette liquid [27, 28].

The solvent extraction method is the most widely used, and there are two main strategies: one involves using water to extract free nicotine from the sample and subsequently employing an appropriate organic solvent to extract free nicotine from the water for determination [24, 29–34]. The other approach involves initially performing organic solvent extraction and then utilizing water to wash and purify the organic extract [35–38]. When these two methods are employed, the introduction of water alters the original distribution of nicotine forms in the sample, leading to an adverse effect and a low recovery rate [15, 17, 25, 39].

Recently, Yang J. et al. proposed a new analytical method for the direct extraction of free nicotine in tobacco using only one solvent [25]. In their method, sieved tobacco powder (including tobacco leaves, cut tobacco, cigar, wrapper, filler, and binder) was directly added to cyclohexane. After static extraction for 24 h, gas chromatography-mass spectrometry (GC-MS) analysis was conducted. No water was used for the extraction and purification. After a comprehensive investigation and verification, it was determined that this method is simple and avoids the impact of aqueous solutions on the original distribution of nicotine forms. Moreover, the coexistence of protonated nicotine in tobacco does not affect the determination.

This paper explores the use of a direct solvent extraction method based on cyclohexane to determine the free nicotine content in the total particulate matter of mainstream cigarette smoke (TPM-o-MCS). The static extraction method was modified to shaking-based extraction, and the extraction time was reduced from 24 to 2 h. Given the high free nicotine content in TPM-o-MCS, the split ratio at the sample inlet was optimized to 250 : 1. Several additional experiments were conducted to analyze and investigate the underlying mechanism of this method.

## 2. Materials and Methods

### 2.1. Reagents and Instruments

A standard solution of nicotine in isopropyl alcohol (9.6 mg/mL) was purchased from Tianjin Alta Scientific Co., Ltd. (Tianjin, China). Cyclohexane (CAS no. 110-82-7, high-performance liquid chromatography grade) was obtained from Thermo Fisher Scientific (Fair Lawn, NJ, USA). Anethole (CAS no. 4180-23-8, 98.5%) was purchased from Beijing Bailingwei Technology (Beijing, China).

Nicotine was analyzed using an automated 20-port rotary smoking machine (RM200A; Borgwaldt KC GmbH, Hamburg, Germany) with a 92-mm glass fiber Cambridge pad (200 pieces per packet), a Milli-Q water purification system (Millipore, Burlington, MA, USA), a speed-adjusting oscillator (HY-8; Guohua Electric Appliance, Changzhou, China), an ultrasonic cleaner (8510E-DTH; Branson Ultrasonics, Sterling Heights, MI, USA), a DL50 titrator, and an AX504 electronic balance with a sensing accuracy of 0.0001 g (Mettler–Toledo, Greifensee, Switzerland). GC-MS analysis was conducted using helium (≥99.999%, by volume; Sichuan Messer Gas Products, Chengdu, China) and 0.2-*μ*m syringe filters (nylon 66, Tianjin Jinteng Experimental Equipment Co., Ltd., Tianjin, China).

### 2.2. Methods

#### 2.2.1. Standard Solution

Anethole (0.50 g, accurate to 0.1 mg) was dissolved in 50 mL of cyclohexane to form an internal standard solution with a concentration of 10.0 mg/mL. Aliquots of 0.25, 0.50, 0.75, 1.00, and 1.25 mL of the standard nicotine solution (9.6 mg/mL) were separately added to 50 mL volumetric flasks. Each flask was then spiked with 0.5 mL of the anethole internal standard solution. Finally, each volumetric flask was filled to the mark with cyclohexane to generate nicotine standard solutions with concentrations of 48.0, 96.0, 144.0, 192.0, and 240.0 *μ*g/mL, corresponding to the above aliquots.

#### 2.2.2. Preparation of Samples

Cigarette samples were selected according to cigarette weight and draw resistance requirements. The selected samples were then preconditioned at (22 ± 2)°C and at a relative humidity of (60 ± 5)% for 48 h. The particulate matter of mainstream cigarette smoke was collected using an automated 20-port rotary smoking machine. Smoking was conducted following the specifications outlined in the International Standard Organization (ISO) Standard 4387, 2019. Twenty cigarettes were smoked using a 92 mm fiber pad with a puff volume of 35 mL, puff duration of 2 s, and a puff interval of 60 s. After smoking, the pad was transferred into a 250 mL extraction bottle. Then, 100 mL of the anethole solution (100 *μ*g/mL in cyclohexane) was added to the bottle, and the mixture was shaken for 2 h at 160 rpm. The resulting mixture was filtered using a 0.2 *μ*m syringe filter (nylon 66), and the filtrate was analyzed via GC-MS.

#### 2.2.3. GC-MS Analysis

Nicotine was analyzed using a 7890B/5977BGC-MS system equipped with an HP-5MS UI column (30 m × 0.250 mm × 0.25 *μ*m; 5% phenyl-methylpolysiloxane; Agilent, Santa Clara, CA, USA).

The GC analysis was performed under the following conditions: helium was used as the carrier gas, with a split ratio of 250 : 1 and a flow rate of 1.0 mL/min. The septum purging rate was 3 mL/min, and a sample volume of 1 *μ*L was injected into the system at a sample inlet temperature of 250°C. The GC oven temperature was initially set at 45°C for 1 min, then increased to 280°C at the rate of 20°C/min, and finally held at 280°C for 2.25 min.

Selected ion monitoring (SIM) was employed, and quantification was based on the internal standard method. The quantitative ion for nicotine was *m*/*z* 84, and the qualitative ions were *m*/*z* 133, 161, and 162. For the internal standard, anethole, the quantitative ion was *m/z* 148, and the qualitative ions were *m/z* 77, 117, and 147. The mass spectrometer was operated in the positive ion mode with an ionization energy of 70 eV. The source temperature was set at 230°C, and the quadrupole temperature was set at 150°C. The transmission line temperature was 280°C, and a solvent delay of 7.0 min was applied.

The solvent delay time of 7 min can be reduced. The retention time of cyclohexane is ∼2.3 min. The authors found that the solvent delay can be set to 2.5 min if needed. To extend the service life of the mass spectrometer detector, the authors set the solvent delay time to 7 min.

## 3. Results

### 3.1. Investigation of Oscillation Time

The effect of shaking-based extraction was investigated as described in Section 2.2.2. Twenty sorted and preconditioned cigarettes were smoked, and the Cambridge pad containing the particulate matter was placed into a 250 mL extraction bottle. Subsequently, 100 mL of anethole solution (100 *μ*g/mL in cyclohexane) was added. The free nicotine in the pads was extracted via oscillation for different durations (Table 1) and analyzed via GC-MS. Table 1 shows that complete extraction of free nicotine from the particulate matter was achieved within 90–120 min. The extraction efficiency and precision were optimal at a shaking duration of 2 h; thus, this duration was adopted for the experiments. In the study by Yang et al., a shaking duration of 3 h and ultrasonic oscillation extraction for 2 h did not result in the complete extraction of free nicotine from tobacco powder, likely owing to the weak penetration of cyclohexane into plant cells [25]. However, the particulate matter of cigarette smoke consists of a nonsolid viscous liquid, primarily composed of tar, which differs from tobacco powder. When captured by the Cambridge pad, the particulate matter is loosely adsorbed or trapped, and the free nicotine molecules are loosely bound to other components in the analytes. Despite the weak penetration of cyclohexane, its solubility for free nicotine is excellent. Therefore, the free nicotine can be completely extracted in a short time through a simple shaking method.

The authors abandoned the attempt to extract free nicotine from cigarette smoke using the ultrasonic oscillation method, because, compared with normal chemical bonds, the hydrogen bonds that form the protonated nicotine are weaker and more likely to break. The cavitation effect of ultrasonic oscillation extraction will produce a strong transient high temperature and high pressure in the local part of the liquid to be measured, which has the risk of transforming the protonated nicotine into free nicotine [25, 40]. During extraction via ultrasonic oscillation, the cavitation effect causes bubbles or voids to expand to a certain critical value under high sound pressure conditions, so that the working frequency of ultrasound becomes infinitely close to or equal to the oscillation frequency of bubbles or holes, resulting in a forceful collapse of bubbles or holes in the compression stage and the release of a large amount of energy. This instantaneously results in high temperatures and pressures around bubbles or holes. Under such high-temperature and high-pressure conditions, the movement speed of the extracted molecules in the liquid medium is accelerated, which may cause the hydrogen bond in the protonated nicotine molecules to break and form free nicotine.

The ultrasonic pressure during extraction via the ultrasonic oscillation method will also endow the extracted particles and the solvent molecules with different acceleration speeds, making the speed of the extracted particles much lower than that of the solvent molecules, resulting in a dislocation effect between the particles and molecules. This dislocation effect can disrupt certain chemical bonds in the extracted molecules. Compared with normal chemical bonds, the hydrogen bonds forming protonated nicotine are weaker and easier to break. Thus, there is a risk that the dislocation effect will result in the conversion of the protonated nicotine into free nicotine during extraction [40].

In addition, the ultrasonic oscillation method has a thermal effect [40]. When the ultrasonic wave propagates in the liquid medium, its energy is continuously absorbed by the medium, and the absorbed ultrasonic energy is converted into heat energy, so that the temperature of the liquid medium continues to rise. This effect also poses the risk of converting the protonated nicotine into free nicotine. To explore the thermal effect of ultrasonic oscillation extraction, experiments were conducted, and the results are shown in Table 2. As presented in the table, when the ultrasonic oscillation method is used for extraction, the temperature of the extraction solution increases by 18°C after 30 min, and after 1 h, it increases by 27°C. The impact of this effect should not be overlooked in the analysis.

Exposure to higher temperatures can lead to the conversion of protonated nicotine into free nicotine, [41] and this phenomenon has been exploited. For example, in the International Standard Organization (ISO) Standard 10315, 2000, isopropyl alcohol extraction and gas chromatography were used to determine the total nicotine in TPM-o-MCS. A sample inlet temperature of 250°C was used. The principle of this method is that when the sample enters the inlet, at the temperature of 250°C, the protonated nicotine will be instantaneously transformed into free nicotine, and then the free nicotine will enter the gas chromatographic column together with the existing free nicotine. This result can be verified from the study conducted by Hu et al. [10]. Hu et al. used 100 mL of 0.025 mol/L H_2_SO_4_ to extract the total nicotine content in TPM o-MCS (i.e., all forms of nicotine were converted into protonated nicotine before analysis), and then the nicotine content was analyzed via ion chromatography. Their method was employed to determine the total nicotine in two certified reference cigarettes (CRCs) provided by the China Tobacco Standardization Center, denoted as CRC-A and CRC-B, and the measured values were 0.588 mg/cig and 0.942 mg/cig, respectively. The certified values of total nicotine in TPM-o-MCS for CRC-A and CRC-B were determined using ISO Standard 10315, 2000, as 0.60 mg/cig and 1.00 mg/cig, respectively. The analysis results of these two methods are consistent, which indicates that the protonated nicotine in TPM-o-MCS can be instantaneously transformed into free nicotine when the sample is injected at the inlet of the gas chromatograph [10].

The extraction of free nicotine from TPM-o-MCS via the ultrasonic oscillation method was also assessed (Table 3). As can be seen from Table 3, the measured value of free nicotine in TPM-o-MCS reached 0.655 mg/cig 20 min after extraction, but the measured value gradually increased over time thereafter. This indicates that when the free nicotine in TPM-o-MCS was extracted via the ultrasonic oscillation method, owing to the effects of cavitation, dislocation, and thermal effects during ultrasonic oscillation extraction, part of the protonated nicotine was converted into free nicotine during extraction.

Owing to the abovementioned reasons, to reduce the risk of conversion of protonated nicotine into free nicotine in the pretreatment stage, the author abandoned the attempt to extract free nicotine using the ultrasonic oscillation method.

### 3.2. pH Value of Cambridge Pad and Its Effects on Determination of Free Nicotine Content

Four 250-mL extraction bottles were used for this experiment. First, 100 mL of deionized water was added to the first two bottles, and 100 mL of deionized water and one Cambridge pad were added to the last two bottles. The bottles were then subjected to oscillation at 160 rpm for 2.0 h. The pH values of the solutions were determined using a DL50 titrator, and the recorded values were 7.27, 7.11, 8.26, and 8.17. These results indicate that the Cambridge pad was weakly alkaline. Standard solutions of low, medium, and high concentrations of free nicotine were prepared and added to 250 mL extraction bottles containing one blank Cambridge pad. The bottles were then subjected to oscillation at 160 rpm for 2.0 h before GC-MS analysis. The recovery percentages of the blank standard additions for the low, medium, and high concentrations of free nicotine were 101.1%, 105.4%, and 105.6%, respectively (Table 4). These experimental results show that the Cambridge pad had no significant impact on the determination of free nicotine content.

The maximum weight of the total particulate matter that can be captured by the Cambridge filter pad is specified in ISO Standard 4387: Glass fiber filter pads of 92 mm diameter can retain up to 600 mg of the total particulate matter. At present, if smoking is performed in accordance with the requirements specified in ISO Standard 4387, 2019, the weight of the total particulate matter will generally not exceed 400 mg. In this study, smoking was also conducted following the specifications outlined in ISO Standard 4387, 2019. We determined 38 commercially available cigarettes, and the weight of the total particulate matter ranged from 146.4 mg to 294.2 mg. Therefore, there is no risk of overloading the Cambridge filter pad.

### 3.3. Method Validation

#### 3.3.1. Standard Curves and Detection Limits

The nicotine standard solution was prepared with five concentrations, and the experiment was conducted using various split ratios at the sample inlet. The regression analysis of the standard curves was performed under different split ratios and fitting conditions, and the results are shown in Table 5. Within the concentration range of the experiment, the quadratic fitting method showed the best correlation for the standard curves under different split ratios. The square of the correlation coefficient (*R*^2^) for the quadratic fitting curves exceeded 0.9997. The second-best method was the second-order natural logarithm method, which also exhibited good correlation, except under the split ratio of 150, where the *R*^2^ value was higher than 0.9998. This observation can be attributed to the fact that as the split ratio decreased or the concentration of the standard solution increased, the amount of free nicotine entering the detector increased. Consequently, the relationship between the concentration of the standard solution and the chromatographic peak area followed a curve rather than a straight line. In particular, at lower split ratios and higher concentrations, the peak area response factors for different concentrations of free nicotine showed significant variations. In contrast, at higher split ratios and lower concentrations, the peak area response factors for different concentrations of free nicotine exhibited minimal differences. Therefore, within a narrow concentration range, linear regression may provide a good fit. However, for a wider concentration range of 48.0–240.0 *μ*g/mL, the quadratic fitting or second-order natural logarithm method is more suitable [25]. After a comprehensive analysis, [42] a split ratio of 250 : 1 and the quadratic fitting method were adopted for the experiment.

In subsequent studies, more than 50 cigarette samples were tested. The content of free nicotine in TPM-o-MCS ranged from 0.350 to 0.780 mg per cigarette (mg/cig), and the concentration of the sample solution during GC-MS analysis ranged from 70.0 to 156.0 *μ*g/mL. These values fell within the concentration range of the standard solution used in the experimental method (48.0–240.0 *μ*g/mL). In all measurements, the *R*^2^ values for the standard curve were higher than 0.999, indicating excellent linearity of the experimental method.

The determination of nicotine content was conducted via step-by-step dilution, and the limit of detection (LOD, signal-to-noise ratio = 3) of the experimental method was determined as 0.56 *μ*g/mL (equivalent to 2.8 *μ*g per cigarette, *μ*g/cig). The limit of quantitation (signal-to-noise ratio = 10) was found as 1.8 *μ*g/mL (equivalent to 9.1 *μ*g/cig). Among the dozens of tested cigarette samples, the lowest content of free nicotine in TPM-o-MCS was 0.350 mg/cig. At this time, the concentration of the sample solution during GC-MS analysis was 70.0 *μ*g/mL. Even when the content of free nicotine in TPM-o-MCS was as low as 0.1 mg/cig, the concentration of the sample solution in GC-MS analysis was still 20 *μ*g/mL, which met the requirements for quantitative analysis.

**Figure 2:**
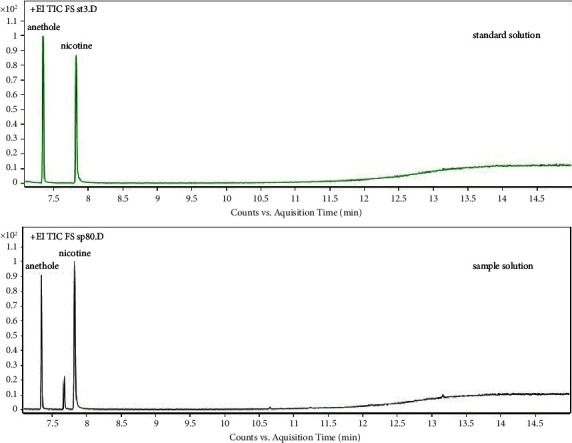
GC-MS chromatogram for FS with a delay of 7.0 min. EI: electron ionization; GC-MS: gas chromatography-mass spectrometry; TIC: total ion current; and FS: full scan.

**Figure 3:**
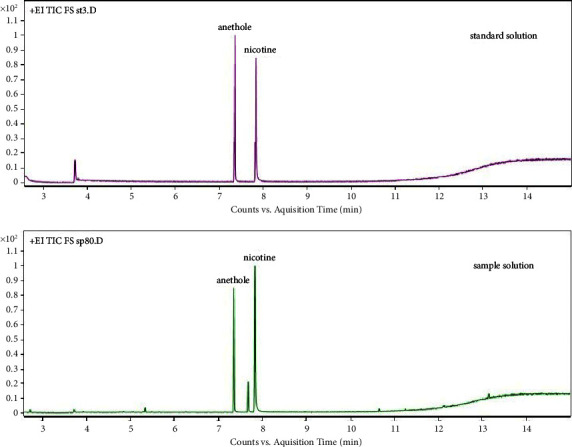
GC-MS chromatogram for FS with a delay of 2.5 min. EI: electron ionization; GC-MS: gas chromatography-mass spectrometry; TIC: total ion current; and FS: full scan.

**Figure 4:**
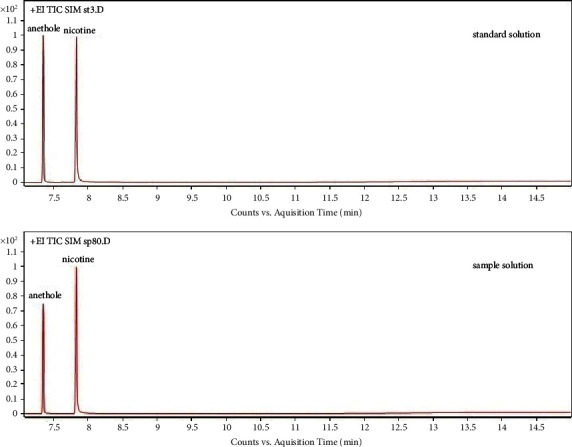
GC-MS chromatogram for SIM. EI: electron ionization; GC-MS: gas chromatography-mass spectrometry; TIC: total ion current; and SIM: selected ion monitoring.

Standard solution and cigarette samples were analyzed via selected ion monitoring (SIM) and a full scan (FS). The chromatograms of the FS of a standard solution and a cigarette sample with a solvent delay of 7 min are shown in Figure 2, and the chromatogram with a solvent delay of 2.5 min is shown in Figure 3. As shown in Figures 2 and 3, there were more other components in the chromatogram for the total ion current (TIC) in FS, particularly between nicotine and anisole, with an unknown large chromatographic peak. However, in the quantitative analysis, because the SIM method was used (Figure 4), chromatographic peaks that were not of interest to the authors did not appear. In addition, complete baseline separation was achieved, and both nicotine and anethole exhibited excellent peak shapes (Figure 4).

#### 3.3.2. Standard Addition Recovery and Precision

After smoking, the Cambridge pad from 20 sorted and preconditioned cigarettes was placed into a 250 mL extraction bottle, and 100 mL of anethole solution (100 *μ*g/mL in cyclohexane) was added. The free nicotine in the pad was then extracted and analyzed via GC-MS following the procedures described in the Methods section. Five parallel measurements were performed. The relative standard deviation (RSD) from the experimental method ranged from 1.7% to 3.4% (Table 6), indicating the high precision of the experimental method. More than 50 cigarette samples were analyzed, and the relative deviations of two parallel samples during the analysis were all less than 6.0%, demonstrating the good repeatability of the experimental method.

The accuracy of the method was verified by determining the recoveries of spiked samples. The experimental method involved selecting a brand of commercial cigarettes and preparing 20 parallel experimental samples, with each sample containing 20 sorted and preconditioned cigarettes. These 20 samples were divided into four groups (labeled A, B, C, and D), with five parallel samples in each group. The average value of free nicotine content in group A was taken as the base value. For cigarette samples from groups B, C, and D, 0.50, 0.80, and 1.10 mL of nicotine standard solution with a concentration of 9.6 mg/mL was added to a 250-mL extraction bottle after cigarette smoking, respectively. The extraction of free nicotine and GC-MS analysis were then conducted following the procedures described in the Methods section. The recoveries of the low-concentration (group B), medium-concentration (group C), and high-concentration (group D) samples were 96.5%, 96.2%, and 102.5% (Table 6), respectively. These results indicate that the experimental method exhibited good recovery rates. The determined value of the free nicotine of the spiked samples (group D) was 186.7 *μ*g/mL, falling within the linear range of the standard solution (48.0–240.0 *μ*g/mL). This indicates that the linear range of the experimental method fully satisfies the requirements of the determination.

### 3.4. Testing of Real Cigarette Samples

The experimental method and the standard method ISO 10315: 2000 were used to determine the contents of free nicotine and total nicotine in TPM-o-MCS from 38 cigarette samples (Table 7). In the standard method, the sorted and preconditioned cigarettes were smoked using an automated 20-port rotary smoking machine, and the Cambridge pad was then extracted with 50 mL of isopropyl alcohol to measure the total nicotine content via GC analysis. Anethole was used as the internal standard for quantitative determination.

In Table 7, 33 of the cigarette samples are Virginia-type cigarettes (VTCs). The reason why there are more cigarettes of this type is that VTCs are one of the most widely sold tobacco products in the world. In China, such tobacco products currently dominate in terms of production and consumption. The primary objective for selecting these commercially available cigarettes was to examine the repeatability and precision of the developed method. Owing to the convenience of purchase, more cigarettes of this type were obtained. These tested samples showed a free nicotine content ranging from 0.376 to 0.716 mg/cig, with an average of 0.540 mg/cig. The relative deviation of parallel samples during the analysis ranged from 0.1% to 5.6%, demonstrating good repeatability of the experimental method (Table 7). The ratio of free nicotine to total nicotine in the tested samples varied from 39.1% to 88.8%, with an average of 61.0%.

### 3.5. Discussion

Accurate measurement of total nicotine content in tobacco and cigarette smoke and the proportions of free and protonated nicotine in total nicotine are crucial for assessing the effect of smoking on overall health, smoking addiction, the tar content of cigarettes, and the sensory quality of cigarette smoke. The authors of this study aimed to identify a suitable analytical method for the accurate determination of free nicotine and total nicotine content in TPM-o-MCS. Measuring total nicotine is relatively easy, but detecting free nicotine poses significant challenges. Consequently, many cigarette manufacturers and research institutions primarily focus on testing total nicotine in tobacco.

Currently, the main methods for detecting free nicotine in tobacco include pH calculation, HS-SPME, NMR spectroscopy, and solvent extraction. However, each approach has its limitations and drawbacks, which restrict their widespread application. The pH calculation method is typically disregarded owing to its drawbacks. This method involves measuring the pH value of a solution formed by dissolving tobacco in water. Tobacco contains various acidic components, alkaline components, and other components that, together with nicotine, contribute to the pH of the solution. However, the introduction of water and other acidic or alkaline components in tobacco can alter the original form of nicotine, rendering the pH calculation method unreliable. Thus, the calculated content of free nicotine in such a solution and at a specific pH value may not accurately represent the actual amount of free nicotine [5, 15, 17]. In addition, the Henderson–Hasselbalch equation, commonly used for calculating free nicotine content, is applicable only to dilute aqueous solutions containing nicotine. Therefore, this method is inaccurate for calculating the free nicotine content in tobacco [5].

HS-SPME is a novel pretreatment method for separating free nicotine and protonated nicotine according to their differences in volatility. However, the drawback of this method is that it is challenging to determine whether free nicotine and protonated nicotine can be completely separated during the headspace separation process. This difficulty arises because, during the creation of the standard curve, the standard solution of free nicotine is applied to the surface of the sample (e.g., sieved tobacco powder), where the combination of nicotine molecules and the matrix is relatively loose. The free nicotine molecules in the sample being tested are located in the vacuoles of tobacco cells, and the combination of nicotine molecules and the matrix is very tight [25]. During headspace separation, the nicotine molecules in the analytes are less likely to volatilize to the gas phase compared with those in the standard solution. To enhance the volatilization of free nicotine into the gas phase, longer headspace separation times or higher temperatures are required. However, free nicotine is a semivolatile component, and tobacco contains numerous highly volatile components such as acetic acid, formic acid, and ammonia. These volatile components will also enter the gas phase at higher temperatures, altering the original acidic or alkaline environment of nicotine molecules in tobacco and affecting the distribution of nicotine in the analytes [20, 22, 23]. Bao et al. employed HS-SPME for the determination of free nicotine content in the total particulate matter of reference cigarette 2R4F and Canadian Monitor 8. The precision of this method ranged from 12.8% to 16.3% (*n* = 9), indicating a need for improvement in precision [21].

NMR spectroscopy involves the use of the NMR spectra of substances to determine the free nicotine content in tobacco. It allows for the direct determination of liquid samples in their original state, providing the advantages of “nondestructive” and “native” analysis [15, 16]. However, this method also has significant shortcomings. First, as an absorption spectroscopy technique, NMR lacks the chromatographic separation capability of other analytical methods. Consequently, the determination of analytes requires pure, high-purity materials or mixtures with relatively simple matrices. For tobacco, which is typically a complex system comprising thousands of components, NMR spectroscopy is not suitable. During the application of NMR spectroscopy to determine free nicotine content, ^1^H NMR detects hydrogen from all components in tobacco. The -CH_3_ peak regions of nicotine and other chemical components may overlap, leading to reduced selectivity and resolution and thus poorer detection limits and baselines [15]. Second, NMR spectroscopy primarily detects liquid or liquid-like analytes. The application of this method is mainly restricted to samples with relatively simple matrices and minimal interference, such as electronic cigarette liquid and its aerosol, [1, 17] and aerosols from heat-not-burn tobacco products [18]. Barsanti et al. utilized two-dimensional NMR spectroscopy (2D HMQC ^1^H/^13^C) to determine free nicotine content in TPM-o-MCS from conventional cigarettes. During the analysis, all spectra were acquired within 4 h at a temperature of 40°C [16]. However, potential changes or transformations in the sample's existing form during such a prolonged analysis at high temperatures are a concern. Furthermore, during NMR analysis, test results can vary significantly among different studies or even for the same study on different subbrands of the same type of sample. For instance, Whidby et al. found that the proportion of free nicotine to total nicotine in TPM-o-MCS ranged from 78% to 96% [19]. Barsanti et al. tested three hard-pack cigarettes and obtained the proportions of free nicotine to total nicotine as 6%, 7%, and 81% [16]. Moreover, the utilization of NMR spectrometry-based analytical methodologies is hindered by limited access to complex and expensive equipment. Therefore, the authors decided not to use this method to determine the free nicotine content in TPM-o-MCS.

Traditional solvent extraction, followed by chromatographic separation, is generally easy to execute and offers good detection limits and precision. Thus, solvent extraction methods are widely employed in the determination of free nicotine content in tobacco. Currently, there are two primary solvent extraction methods. One method involves extracting free nicotine from tobacco using water and subsequently extracting it from the water using an appropriate organic solvent for analysis. This method relies on the fact that free nicotine and certain protonated nicotine compounds are initially extracted into the water. When both aqueous and organic phases are present, protonated nicotine remains in the aqueous phase because it readily dissolves in water, while free nicotine can be selectively extracted as it is more soluble in the organic phase, thus achieving purification [24, 29–34]. Another method is to extract free nicotine with an organic solvent first and then purify the organic extract with water for analysis. During the analysis, the free nicotine and some nicotine salts combined with organic acid were first extracted into the organic solvent, and then, water was added to extract the protonated nicotine from the organic solvent for purification [35–38]. Since free nicotine has good solubility in both aqueous and organic phases, the conversion of nicotine molecules between the free and protonated states is affected by the pH of the solution, and there are many other acidic and alkaline components in tobacco. Therefore, the original distribution of nicotine in tobacco will be altered by the two extraction methods, leading to poor recovery [25, 39].

El-Hellani et al. studied the determination of free nicotine content in e-cigarette liquid by dissolving 300 *μ*L of the liquid to be measured in 5 mL water, adding 5 mL toluene, shaking for 30 min, stratifying, and then detecting the nicotine content in toluene, which was recorded as the content of free nicotine to be measured [29]. Since free nicotine had a good solubility in toluene and water, the sample also contained other acids, alkaline, and other components. After the analytes dissolved in water, the free nicotine and protonated nicotine in tobacco were transformed under the pH condition to form an equilibrium. Then, owing to the introduction of toluene, the two forms of nicotine underwent complex conversion in the toluene phase and the water phase to form a new equilibrium. Thus, the content of the free nicotine in the toluene phase after conversion was measured. To investigate whether conversion occurs during the determination of free nicotine, Duell et al. experimented with D_2_O and found that the free nicotine in the JUUL “crème brulee” e-cigarette liquid was fully transformed into monoprotonated nicotine after dilution with D_2_O (dilution ratio 5 : 1, by volume) [17]. This led the authors to decide to experiment with other solvent extraction methods.

Yang et al. proposed a new analytical method for the direct extraction of free nicotine in tobacco using only one solvent. The sieved tobacco powder was added directly to cyclohexane, which was extracted for 24 h and analyzed via GC-MS (no water was added for purification) [25]. This method is simple and avoids the effect of an aqueous solution on the original distribution of nicotine in tobacco, and the coexistence of protonated nicotine in analytes has no effect on the determination.

After careful consideration, we believe that Yang et al.'s method has certain advantages and is reasonable. First, free nicotine can penetrate and pass through the lipid layer of cell tissues, and it exhibits good solubility in both aqueous and organic phases. In contrast, protonated nicotine cannot penetrate the lipid layer and can only dissolve in the water phase. Therefore, solvents such as water, isopropyl alcohol, propylene glycol, glycerol, acetone, and methyl t-butyl ether can dissolve both the free and protonated forms of nicotine, [14] while cyclohexane can only dissolve the free form of nicotine [5, 7]. Second, the transformation of nicotine between the free and protonated nicotine forms can occur between two solvents. For instance, when the tobacco sample is dissolved in immiscible toluene and water, the forms of nicotine in the analytes will transform after reaching equilibrium. The resulting distribution is determined by the distribution coefficient of free nicotine in toluene and water, along with other factors. Similarly, nicotine forms can undergo transformation in a solvent that can provide hydrogen ions. For example, when tobacco powder is dissolved in water at a specific pH value, the distribution of free nicotine in the analytes will transform into the distribution corresponding to that pH value after an equilibrium is reached. In addition, nicotine forms can undergo transformation in a solvent that can alter the presence of hydrogen ions in the nicotine molecules. For instance, in solvents such as acetone or isopropyl alcohol, the presence of nicotine in the sample can also be altered owing to the influence of polar oxygen and hydrogen bonding. Cyclohexane is an inert solvent with very weak polarity. It cannot provide hydrogen ions and does not easily modify the binding state of hydrogen ions in the nicotine molecule, particularly in the vicinity of the nitrogen element. As a result, the original form of nicotine in the sample is less likely to undergo changes when dissolved in cyclohexane [25]. Third, tobacco powder and cigarette smoke particulate matter are not in a liquid state, and free nicotine and protonated nicotine do not exist in a uniform dynamic equilibrium within the sample. Rather, they are relatively independent. When the sample is dissolved in cyclohexane, which selectively dissolves only the free nicotine, the free nicotine enters the organic phase, while the protonated nicotine remains outside the organic phase. This approach minimizes disturbances to the sample and provides measurement results that better reflect the actual distribution of free nicotine in the analytes. Finally, compared with HS-SPME and NMR spectroscopy, the analytical method involving solvent extraction, followed by chromatographic separation detection, is generally easier to perform. It offers better precision and detection limits and exhibits fewer interferences during the instrumental detection stage (owing to the separation function of chromatographic analysis). Therefore, after careful consideration, this method was used for the determination of free nicotine content in TPM-o-MCS.

Unlike the determination of free nicotine content in tobacco, the analysis of free nicotine content in TPM-o-MCS involves the use of the Cambridge filter pad. The pH value and adsorption properties of the pad may impact the determination process. To address this, the authors conducted tests to assess the pH value of the Cambridge pad and the blank standard addition recovery of the method. The results indicated that the pH value of the Cambridge pad ranged from 8.17 to 8.26, which did not alter the existing form of free nicotine in TPM-o-MCS. Furthermore, the blank standard addition experiments demonstrated recoveries of 101.1%–105.6%, suggesting that the pH value and adsorption properties of the Cambridge pad did not affect the determination of free nicotine content.

In the experiments conducted by Yang et al., the determination of free nicotine content in tobacco involved a 24 h static extraction process. This extended duration was necessary because cyclohexane featured weak penetration into plant cells, and the free nicotine present in tobacco powder could not be fully extracted via ultrasonic oscillation for 2 h or shaking for 3 h. Hence, the 24 h static extraction method was employed. The particulate matter of cigarette smoke, unlike tobacco powder, is a highly viscous liquid predominantly composed of tar. When the particulate matter was captured by the Cambridge pad, a significant portion of it was loosely adsorbed on the pad's surface. The binding between free nicotine and other components in TPM-o-MCS and the interaction between free nicotine and the Cambridge pad were relatively weak. Consequently, the free nicotine was easily dissolved by cyclohexane, allowing for its complete extraction by shaking for 2 h. Furthermore, Yang et al. employed a small split ratio (20 : 1) at the sample inlet during GC-MS analysis to determine the free nicotine content in tobacco. However, owing to the high concentration of free nicotine in TPM-o-MCS, the small split ratio was found to be unsuitable. After conducting numerous experiments and investigations, the authors decided not to increase the dilution steps during the pretreatment stage. Instead, they opted for a higher split ratio (250 : 1) to simplify the operational steps and improve the method's performance. The experimental results demonstrated enhanced precision, lower LOD, improved linearity, and higher standard addition recovery, all of which adequately satisfy the requirements for determining free nicotine content in TPM-o-MCS.

## 4. Conclusions

Currently, there are various methods for detecting free nicotine in tobacco, including pH calculation, NMR spectroscopy, HS-SPME, and traditional solvent extraction. However, each method has its limitations, which restrict its application. In this study, according to the traditional solvent extraction method, the content of free nicotine in TPM-o-MCS was determined via direct oscillation-based extraction using cyclohexane. Compared with the traditional two-phase solvent extraction, the experimental method offers several advantages. It is simple to perform and avoids the influence of aqueous solutions on the original distribution of nicotine forms in the sample. In addition, the coexistence of protonated nicotine in tobacco does not affect the determination. Compared with HS-SPME and NMR spectroscopy, the proposed method, which involves solvent extraction, followed by chromatographic separation and instrumental detection, demonstrates better precision and detection limits and reduced interference during instrumental detection. Hence, it is suitable for determining the content of free nicotine in TPM-o-MCS.

The standard addition recoveries during the conducted experiment ranged from 96.2% to 102.5%. The LOD was 2.8 *μ*g/cig, and the correlation coefficient (*R*^2^) for the quadratic regression of the standard curve was >0.999. The RSD for parallel samples ranged from 1.7% to 3.4% (*n* = 5), meeting the requirements for the determination of free nicotine content in TPM-o-MCS. Analysis of 38 cigarette brands revealed that the content of free nicotine in TPM-o-MCS ranged from 0.376 to 0.716 mg/cig, with an average of 0.540 mg/cig, and the free nicotine accounted for 39.1%–88.8% of the total nicotine content.

## Figures and Tables

**Table 1:** Oscillation time during extraction.

Time (min)	Free nicotine concentration (*μ*g/mL)	RSD (%, *n* = 5)
60	113.0	1.7
90	115.0	2.4
120	117.6	1.2
150	116.8	1.9
180	112.5	4.2

Rotation speed ∼160 rpm; RSD, relative standard deviation.

**Table 2:** Thermal effect of ultrasonic oscillation extraction.

Time (min)	Temperature (°C)
0	22
30	40
60	49
90	53
99	54

Ultrasonic cleaner output: 44 kHz, 250 W.

**Table 3:** Ultrasonic oscillation extraction with different times.

Time (min)	Determined value (mg/cig)	RSD (%, *n* = 5)
20	0.655	4.2
40	0.718	4.2
60	0.735	1.5
80	0.741	3.0

Sonics: 44 kHz, 250 W; RSD, relative standard deviation; through the constant injection of cold water, the water temperature was maintained at 30°C.

**Table 4:** Blank standard addition recovery of free nicotine.

Blank Cambridge pad (*μ*g/mL)	Free nicotine added (*μ*g/mL)	Determined value (*μ*g/mL)	Recovery (%)
ND	96.0	97.1	101.1
ND	144.0	151.8	105.4
ND	192.0	202.8	105.6

ND, not detected.

**Table 5:** Correlation coefficients (*R*^2^) of the standard curves under different split ratios and fittings.

Split ratios	Quadratic fitting	Monomial	SONL	Linear
50 : 1	0.99977	0.99973	0.99982	0.97762
100 : 1	0.99980	0.99639	0.99991	0.97639
150 : 1	0.99990	0.99504	0.99783	0.98978
200 : 1	0.99987	0.99814	0.99986	0.99260
250 : 1	0.99997	0.99831	0.99996	0.99335

SONL, second-order natural logarithm.

**Table 6:** Standard addition recovery and precision of the experimental method.

Base value (*μ*g/mL)	Amount added (*μ*g/mL)	Determined value (*μ*g/mL)	Recovery (%)	RSD (%, *n* = 5)
78.5				3.4
78.5	48.0	124.8	96.5	2.8
78.5	76.8	152.3	96.2	1.7
78.5	105.6	186.7	102.5	2.0

**Table 7:** Contents of free nicotine and total nicotine in 38 cigarette samples.

No.	Type	Free nicotine		Total nicotine (mg/cig)	FN/TN (%)
		Conc. (mg/cig)	RD (%)		
1	VTC	0.484	2.3	0.86	56.3
2	VTC	0.480	2.3	0.97	49.5
3	VTC	0.458	0.1	0.82	55.9
4	VTC	0.535	0.4	0.80	66.9
5	VTC	0.499	0.4	0.95	52.5
6	VTC	0.716	5.6	0.89	80.4
7	VTC	0.489	0.3	0.98	49.9
8	VTC	0.465	0.8	1.19	39.1
9	CTC	0.577	3.5	0.78	74.0
10	EFTC	0.512	2.1	0.59	86.8
11	VTC	0.583	1.1	0.99	58.9
12	VTC	0.476	0.1	0.85	55.9
13	CTC	0.426	2.2	0.86	49.5
14	VTC	0.574	1.6	0.97	59.1
15	VTC	0.604	0.9	1.03	58.6
16	VTC	0.429	4.1	1.04	41.3
17	VTC	0.609	0.7	1.04	58.5
18	VTC	0.666	0.3	0.75	88.8
19	VTC	0.579	4.6	1.13	51.2
20	VTC	0.459	2.9	0.91	50.5
21	VTC	0.508	0.6	0.89	57.1
22	VTC	0.445	0.8	0.86	51.7
23	VTC	0.376	0.4	0.77	48.8
24	VTC	0.530	0.8	0.68	78.0
25	VTC	0.494	3.9	0.95	52.0
26	CTC	0.501	2.5	0.84	59.6
27	EFTC	0.466	3.8	0.58	80.3
28	VTC	0.670	0.1	1.07	62.6
29	VTC	0.683	0.7	1.04	65.7
30	VTC	0.640	4.4	1.02	62.8
31	VTC	0.642	4.2	0.94	68.3
32	VTC	0.444	1.2	0.69	64.4
33	VTC	0.666	1.5	1.11	60.0
34	VTC	0.461	2.7	0.70	65.8
35	VTC	0.594	4.5	1.03	57.7
36	VTC	0.558	4.8	0.83	67.2
37	VTC	0.634	2.4	0.87	72.9
38	VTC	0.596	4.7	1.02	58.4

FN/TN, ratio of free nicotine to total nicotine; RD, relative deviation; VTC, Virginia-type cigarette; CTC, cigar-type cigarette; EFTC, exotic flavor-type cigarette.

## Data Availability

The data used to support the findings of this study are available from the corresponding author upon request.
